# Knee donor-site morbidity after mosaicplasty – a systematic review

**DOI:** 10.1186/s40634-016-0066-0

**Published:** 2016-11-03

**Authors:** Renato Andrade, Sebastiano Vasta, Rogério Pereira, Hélder Pereira, Rocco Papalia, Mustafa Karahan, J. Miguel Oliveira, Rui L. Reis, João Espregueira-Mendes

**Affiliations:** 1 Faculty of Sports, University of Porto, Porto, Portugal; 2 Clínica do Dragão, Espregueira-Mendes Sports Centre - FIFA Medical Centre of Excellence, Porto, Portugal; 3 Dom Henrique Research Centre, Porto, Portugal; 4 Orthopaedic and Trauma Department, Campus Biomedico University of Rome, Rome, Italy; 5 Faculty of Health Sciences, University of Fernando Pessoa, Porto, Portugal; 6 Orthopaedic Department, Centro Hospitalar Póvoa de Varzim, Vila do Conde, Portugal; 7 3B’s Research Group–Biomaterials, Biodegradables and Biomimetics, University of Minho, Headquarters of the European Institute of Excellence on Tissue Engineering and Regenerative Medicine, AvePark, Parque de Ciência e Tecnologia, Zona Industrial da Gandra, Barco, 4805-017 Guimarães, Portugal; 8 ICVS/3B’s–PT Government Associate Laboratory, Braga/Guimarães, Portugal; 9 Ripoll y De Prado Sports Clinic FIFA Medical Centre of Excellence, Murcia-Madrid, Spain; 10 Department of Orthopaedic Surgery, Acibadem University, Istanbul, Turkey; 11 Orthopaedics Department of Minho University, Minho, Portugal

**Keywords:** Knee, Donor-site morbidity, Osteochondral, Mosaicplasty, Articular cartilage lesions

## Abstract

**Background:**

Mosaicplasty has been associated with good short- to long-term results. Nevertheless, the osteochondral harvesting is restricted to the donor-site area available and it may lead to significant donor-site morbidity.

**Purpose:**

Provide an overview of donor-site morbidity associated with harvesting of osteochondral plugs from the knee joint in mosaicplasty procedure.

**Methods:**

Comprehensive search using Pubmed, Cochrane Library, SPORTDiscus and CINAHL databases was carried out through 10^th^ October of 2016. As inclusion criteria, all English-language studies that assessed the knee donor-site morbidity after mosaicplasty were accepted. The outcomes were the description and rate of knee donor-site morbidity, sample’s and cartilage defect’s characterization and mosaicplasty-related features. Correlation between mosaicplasty features and rate of morbidity was performed. The methodological and reporting quality were assessed according to Coleman’s methodology score.

**Results:**

Twenty-one studies were included, comprising a total of 1726 patients, with 1473 and 268 knee and ankle cartilage defects were included. The defect size ranged from 0.85 cm^2^ to 4.9 cm^2^ and most commonly 3 or less plugs (averaging 2.9 to 9.4 mm) were used. Donor-site for osteochondral harvesting included margins of the femoral trochlea (condyles), intercondylar notch, patellofemoral joint and upper tibio-fibular joint. Mean donor-site morbidity was 5.9 % and 19.6 % for knee and ankle mosaicplasty procedures, respectively. Concerning knee-to-knee mosaicplasty procedures, the most common donor-site morbidity complaints were patellofemoral disturbances (22 %) and crepitation (31 %), and in knee-to-ankle procedures there was a clear tendency for pain or instability during daily living or sports activities (44 %), followed by patellofemoral disturbances, knee stiffness and persistent pain (13 % each). There was no significant correlation between rate of donor-site morbidity and size of the defect, number and size of the plugs (*p* > 0.05).

**Conclusions:**

Osteochondral harvesting in mosaicplasty often results in considerable donor-site morbidity. The donor-site morbidity for knee-to-ankle (16.9 %) was greater than knee-to-knee (5.9 %) mosaicplasty procedures, without any significant correlation between rate of donor-site morbidity and size of the defect, number and size of the plugs. Lack or imcomplete of donor-site morbidity reporting within the mosaicplasty studies is a concern that should be addressed in future studies.

**Level of evidence:**

Level IV, systematic review of Level I-IV studies.

## Background

Treating full-thickness cartilage lesions of weight-bearing joints still remains a clinical challenge in orthopaedics. The articular cartilage has been described as a highly organized tissue with complex biomechanical properties and considerable durability (Simon & Jackson 2006). However, due to its avascular and hypocellular nature (Gomoll & Minas 2014), the articular cartilage has limited intrinsic capacity for spontaneous healing (Gomoll & Minas 2014; McAdams et al. 2010; Steinwachs et al. 2012). Articular cartilage lesions often cause pain, instability and disability (Heijink et al. 2012; Bedi et al. 2010), and might lead to an early onset of degenerative changes (Bedi et al. 2010; Gomoll et al. 2012). In this sense, orthopedic surgeons have pursuit in the past an approach that can allow achieving the hyaline or hyaline-like repair of articular defects.

The osteochondral autograft transfer (OATS) is based on transfering autologous whole tissue (bone and cartilage), using a single or multiple osteochondral autografts, for delivering genuine hyaline articular cartilage to the defect, aiming the immediate restoration of the joint surface (Krych et al. 2016). László Hangody (Hangody et al. 1997; Hangody & Karpati 1993) in 1992, created the mosaicplasty resurfacing concept, involving the transfer of multiple small-sized, cylindrical osteochondral grafts. This procedure aimed to overcome the limitations and difficulties in repairing focal, full-thickness cartilage lesions of weight-bearing areas of the femoral condyles, patella, and talus. Since then, long-term results have shown promising outcomes (Gomoll et al. 2012; Lynch et al. 2015; Hangody et al. 2010). This technique has been indicated majorly for small-to-medium size focal articular cartilage or osteochondral defects of the weigh-bearing areas of the femoral condyles, patellofemoral joint and talus (Bartha et al. 2006; Hangody & Füles 2003). Indications have grown and the elbow joint has been considered has a potential recipient site (Vezeridis & Bae 2016; Lyons et al. 2015). The derived osteochondral plugs may be suitable for filling deep (>8-10 mm) and/or large osteochondral defects in cases that sandwich strategy (combined autologous chondrocyte implantation and subchondral bone restoration procedure) is not possible (Peterson 2003).

The mosaicplasty surgical procedure has the advantage of transplanting viable hyaline-like tissue matrix and subchondral bone in a single-step procedure (Bedi et al. 2010; Bartha et al. 2006; Hangody & Füles 2003; Mundi et al. 2015; Espregueira-Mendes et al. 2012; Moran et al. 2014). Nevertheless, this technique is restricted by the availability of autologous graft that can be harvested and by the donor-site associated morbidity (Bedi et al. 2010; Moran et al. 2014; Reddy et al. 2007). Furthermore, there is limited evidence on the short and long-term consequences from harvesting bone plugs of asymptomatic joints (LaPrade & Botker 2004; Paul et al. 2009). Herein, it was aimed to characterize and quantify the reported donor-site morbidity associated with the harvesting of osteochondral plugs from the knee joint. It is hypothesized that harvesting osteochondral plugs from the knee joint will result in a considerable rate of donor-site morbidity.

## Methods

### Search strategy

The systematic review of the literature was conducted according to the Preferred Reporting Items for Systematic Reviews and Meta-Analyses (PRISMA) statement, which aims to improve the standard of reporting of systematic reviews and meta-analyses (Liberati et al. 2009). The protocol used was *a priori* registered at the International Prospective Register of Systematic Reviews (PROSPERO) (http://www.crd.york.ac.uk/prospero/; ID: CRD42016032861).

A comprehensive database search using Pubmed, Cochrane Library, SPORTDiscus and Cumulative Index of Nursing and Allied Health (CINAHL) was carried out. We included original articles that assessed the occurrence of morbidity associated with the mosaicplasty surgical procedure. All searches were performed up to October 10, 2016. Two investigators (R.A., S.V.) performed the search independently, and results were confronted to check for overlapping; any disagreement was discussed until consensus was reached, involving the senior authors. The reference list of the most relevant original studies was scanned for additional studies. The search strategy combined the following search terms: mosaicplasty; OATS; “osteochondral autograft”; “osteochondral transfer”; “osteochondral transplant”; morbidity; “donor-site”; harvest; “postoperative complications”.

### Study selection

All the titles and abstracts obtained from the databases were screened for relevant articles. The potential relevant studies identified were retrieved and the respective full text analyzed for their eligibility according the following inclusion criteria: (1) report of knee donor-site related morbidity associated to the mosaicplasty procedures, i.e., to be included, the original study had to report the occurrence of knee morbidity symptoms (not requiring a specific/focused questionnaire); (2) follow-up of, at least, 6 months; (3) inclusion of level I-IV studies; (4) prospective or retrospective studies with a cohort over 10 patients (*n* > 10); (5) human subjects; and (6) English language studies. For exclusion criteria it was determined: (i) other reviews or meta-analyses; (ii) clinical commentaries, expert opinions or technical notes; (iii) single case studies or case series with a cohort bellow 10 participants; (iv) animal studies or basic science; (v) skeletally immature population; (vi) cadaveric studies; (vii) synthetic grafts; (viii) allografts; (ix) mosaicplasty procedures performed as a complementary procedure of other surgical procedures (such as, anterior cruciate ligament (ACL) reconstruction or meniscal transplantation); and (x) all study cohort with use of adhesive patches on the donor-site area.

### Data collection and extraction

The main outcome of interest was the presence of donor-site morbidity after the mosaicplasty surgical procedure. Following the eligibility criteria screening and the determination of the articles to be included, the studies were divided into knee-to-knee or knee-to-ankle mosaicplasty surgical procedures and analyzed based on: (i) sample demographics; (ii) defect characteristics; (iii) method of radiological evaluation; (iv) donor-site for the autologous osteochondral graft; (v) characteristics of the osteochondral plugs; (vi) time until surgery and follow-up; (vii) number of previous surgeries, concomitant procedures and complications; and (viii) description and rate of donor-site morbidity.

### Methodological quality assessment

The methodological quality of the included original studies was assessed through the Coleman methodology score (Coleman et al. 2000) and the level of evidence was accordingly set. The Coleman methodology score assesses the study’s quality of reporting their methodology according ten criteria divided into two sections, resulting in a total score between 0 and 100.

### Statistical analysis

The main outcome is the percentage of donor-site morbidity reported within the included studies. It was calculated the correlation between the donor-site morbidity (%) and the size of the cartilage defect (mm^2^), the number of osteochondral plugs (n) and the size of the osteochondral plugs (mm). The data from the included studies was added into the statistical analysis if the mean of the required variable was reported or able to be calculated. Otherwise, they were not included into the statistical analysis. Once these variables had a non-parametric behavior, the coefficient of the Spearman correlation was used. The statistical analysis was performed through the program Statistical Package for the Social Sciences (SPSS®, IBM, Chicago, IL, USA) v.21.0. The level of significance (p) was set at 0.05 for the hypothesis tests.

## Results

### Study selection

**Fig. 1:**
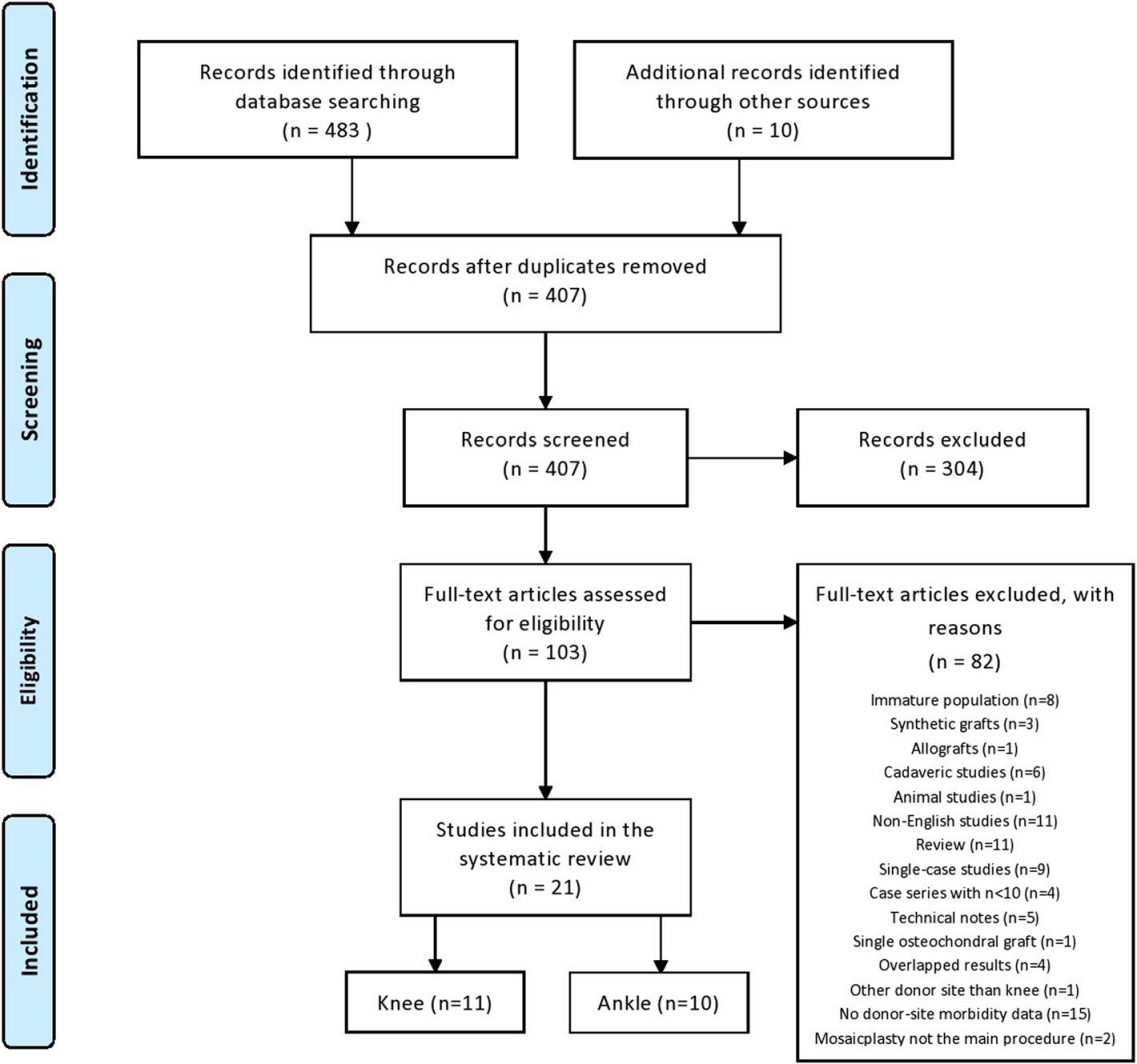
Preferred Reporting Items for Systematic Reviews and Meta-Analyses (PRISMA) flow chart for the database search

The database and hand search yielded 493 titles and abstracts. An example of the search is depicted on Table 1. Duplicated articles were removed and 407 articles were screened based on their title and abstract. A total of 103 full-text articles were screened according the inclusion and exclusion eligibility. Following the full-text screening, 21 original studies (Hangody et al. 2010; Espregueira-Mendes et al. 2012; Reddy et al. 2007; Gudas et al. 2005; Ahmad & Jones 2015; Al-Shaikh et al. 2002; Atik et al. 2005; Baltzer & Arnold 2005; de l'Escalopier et al. 2015; Gautier et al. 2002; Hangody et al. 2001a; Hangody et al. 2008; Jakob et al. 2002; Kim et al. 2012; Kock et al. 2010; Koulalis et al. 2004; Lee et al. 2003; Quarch et al. 2014; Reverte-Vinaixa et al. 2013; Valderrabano et al. 2009; Clavé et al. 2016) were eligible for inclusion in the systematic review, which were further subgrouped into knee and ankle joints. All studies concerning mosaicplasty procedure performed in the upper limb have been excluded based on the initially established criteria, including: immature population (Vezeridis & Bae 2016; Lyons et al. 2015; Nishimura et al. 2011; Iwasaki et al. 2007; Iwasaki et al. 2009; Shimada et al. 2005); single case-study (Zelent & Neese 2005); non-English language (Braun et al. 2012); case series under 10 participants (Han et al. 2012; Tsuda et al. 2005). Search strategy steps and reasons for inclusion can be seen at the PRISMA flow chart (Fig. 1).

**Table 1:** Example of search strategy for Pubmed database

Search	Search term(s)	Results
*#1*	Search mosaicplasty	259
*#2*	Search OATS	4 816
*#3*	Search “osteochondral autograft”	268
*#4*	Search “osteochondral transfer”	29
*#5*	Search “osteochondral transplant”	16
*#6*	Search (#1 OR #2 OR #3 OR #4 OR #5)	5 292
*#7*	Search morbidity	2 200 564
*#8*	Search “donor site”	10 680
*#9*	Search harvest	18 740
*#10*	Search “postoperative complications”	333 798
*#11*	Search (#7 OR #8 OR #9 OR #10)	2 465 489
*#12*	Search (#6 AND #11)	369

### Population characteristics

Characteristics of the sample population and articular cartilage / osteochondral defects from the 21 included original studies are depicted in Table 2. Overall, a total of 1726 patients (1472 and 254 patients underwent knee and ankle mosaicplasty, respectively) with a mean age of 33.2 years and 34.8 years old for the knee and ankle joints cohorts, respectively. The original included studies comprised mostly small sample sizes (between 11 and 30 participants) (Reddy et al. 2007; Gudas et al. 2005; Ahmad & Jones 2015; Al-Shaikh et al. 2002; Atik et al. 2005; Gautier et al. 2002; Kock et al. 2010; Lee et al. 2003; Quarch et al. 2014; Reverte-Vinaixa et al. 2013; Valderrabano et al. 2009; Clavé et al. 2016), a few moderate samples sizes (between 31 and 48 participants) (Espregueira-Mendes et al. 2012; Baltzer & Arnold 2005; de l'Escalopier et al. 2015; Hangody et al. 2001a; Jakob et al. 2002; Kim et al. 2012) and two large scale retrospective studies(303 and 967 participants) (Hangody et al. 2010; Hangody et al. 2008).

**Table 2:** Population and articular cartilage / osteochondral defects characteristics

First author (year)	Population	M : F	Age (years)	Defect location	Defect size mean (range)	Defect classification	Radiological evaluation
Knee							
Atik et al. (2005)	*n* = 12	6 : 6	38	MFC (*n* = 9) LFC (*n* = 1) Patella (*n* = 2)	>1 cm diameter	Outerbridge Grade IV	Arthroscopy
Espregueira-Mendes et al. (2012)	*n* = 31	22 : 9	30.1	MFC (*n* = 22) LFC (*n* = 7) Trochlea (*n* = 1) Patella (*n* = 1)	3.3 cm^2^	ICRS Grade IV	MRI
Gudas et al. (2005)	*n* = 28	19 : 10	24.6	MFC (*n* = 25) LFC (*n* = 3)	2.8 cm^2^	ICRS OCD (*n* = 13) Full-thickness (*n* = 15)	MRI Radiography
Hangody et al. (2008)	*n* = 967	N.R.	N.R.	Femoral condyle (*n* = 789) Patella (*n* = 147) Tibia condyles (*n* = 31)	N.R.	Outerbridge Grade III or IV (66 %) Osteochondral defects (33 %)	MRI Radiography
Hangody et al. (2010)	*n* = 303	N.R.	N.R.	MFC (*n* = 187) LFC (*n* = 74) LTC (*n* = 15) MTC (*n* = 1) Patella (*n* = 18) Trochlear (*n* = 8)	2.8 cm^2^ (1-5) 1.8 cm^2^ (1-4) 1.2 cm^2^ (1-2) 1 cm^2^ 2.4 cm^2^ (1-3) 2.1 cm^2^ (1-3.5)	Outerbridge Grade III or IV (66 %) Shallow osteochondral lesions (33 %)	MRI Radiography
Jakob et al. (2002)	*n* = 42	34 : 18	34	MFC (*n* = 10) LFC (*n* = 5) Patella (*n* = 1) ^b^	4.9 cm^2^ (1.5-16)	ICRS Grade III (*n* = 23) Grade IV (*n* = 29)	MRI
Kock et al. (2010)	*n* = 13	8 : 5	33	MFC (*n* = 10) LFC (*n* = 3)	N.R.	Full-thickness cartilage lesions	Bone scintigraphy
Koulalis et al. (2004)	*n* = 18	12 : 6	36	MFC (*n* = 13) LFC (*n* = 2) Patella (*n* = 3) Trochlear (*n* = 1)	2.5 cm^2^	Outerbridge Grade IV	MRI Radiography
Quarch et al. (2014)	*n* = 16	N.R.	39.7	MFC (*n* = 12) LFC (*n* = 1) Patella (*n* = 3)	4.6 cm^2^	Grade I-IV	MRI
Reverte-Vinaixa et al. (2013)	*n* = 17	12 : 5	35	MFC (*n* = 3) LFC (*n* = 14)	3.4 cm^2^ (1-4)	Outerbridge Grade III/IV	MRI
Clavé et al. (2016)	*n* = 25	20 : 5	28.3	Femoral condyle (*n* = 25)	3.5 cm^2^	ICRS Grade I (*n* = 1) Grade III (*n* = 2) Grade IV (*n* = 22)	MRI
Ankle							
Ahmad and Jones (2015)	*n* = 20	11 : 9	41.3	Anteromedial (*n* = 2) Anterocentral (*n* = 1) Anterolateral (*n* = 3) Centromedial (*n* = 7) Central direct (*n* = 1) Centrolateral (*n* = 2) Posteromedial (*n* = 4)	1.6 cm^2^ (0.7-2.4)	N.R.	CT MRI Radiography
Al-Shaikh et al. (2002)	*n* = 19	6 : 13	32	Medial dome (*n* = 15) Lateral dome (*n* = 3) Both (*n* = 1)	1.2 cm^2^ (0.5-4)	Berndt/Hardy classification Grade I (*n* = 4) Grade II (*n* = 6) Grade III (*n* = 2) Grade IV (*n* = 7)	MRI Radiography
Baltzer and Arnold (2005)	*n* = 43	30 : 13	31.2	Medial (*n* = 27) Lateral (*n* = 14) Central (*n* = 2)	1.7 cm^2^ (up to 3.7 cm^2^)	Outerbridge Grade III or IV	MRI Radiography
Gautier et al. (2002)	*n* = 11	8 : 3	31.9	Medial (*n* = 10) Lateral (*n* = 1)	1.8 cm^2^ (0.7-4.2)	Berndt and Harty Grade II-IV	CT MRI Radiography
Hangody (2001)	*n* = 36	N.R.	27	N.R.	1 cm^2^	Berndt and Harty classification Grade III Grade IV	CT MRI Radiography
Kim et al. (2012)	*n* = 48	34 : 18	48.2	Anteromedial (*n* = 8) Centrolateral (*n* = 15) Posteromedial (*n* = 29)	1.5 cm^2^ (0.5-2.9)	N.R.	MRI Radiography
Lee et al. (2003)	*n* = 17	16 : 1	22.7	Medial (*n* = 16) Lateral (*n* = 2)	1.0 cm^2^ (0.6-4)	Berndt and Harty Grade III-IV	Arthroscopy CT MRI Radiography
de l’Escalopier et al. (2015)	*n* = 37	29 : 8	33	Medial (*n* = 12) Posteromedial (*n* = 14) Lateral (*n* = 11)	0.85 cm^2^ (0.4-2.12)	N.R.	CT MRI Radiography
Reddy et al. (2007)	*n* = 11	5 : 6	29	Medial (*n* = 8) Posteromedial (*n* = 1) Central (*n* = 1) Anterocentral (*n* = 1)	1.3 cm^2^	Full thickness defects	MRI Radiography
Valderrabano et al. (2009)	*n* = 12^a^	8 : 4^a^	43^a^	Medial (*n* = 7) Lateral (*n* = 14)	1.4 cm^2^ (0.5-3.6)	Berndt/Harty classification^a^ Grade IV (*n* = 9) Grade V (*n* = 5)	MRI SPECT-CT

*Legend: MFC* Medial femoral condyle, *LFC* Lateral femoral condyle, *LTC* Lateral tibial condyle, *MTC* Medial tibial condyle, *N.R.* Not reported, *ICRS* International Cartilage Repair Society score, *OCD* Osteochondritis dissecans, *MRI* Magnetic resonance imaging, *CT* Computed tomography, *SPECT-CT* Single-photon emission computed tomography

^a^ Report of the 12 included patients on the follow-up, from a cohort of 21 patients; ^b^ Results reported from the patients with osteochondritis dissecans and localized degeneration; the other articular cartilage lesions came from other reasons, such as, acute trauma or femoropatellar arthrosis

### Articular cartilage / osteochondral defects characteristics

A combined number of 1473 articular cartilage / osteochondral defects on the knee and 268 on the ankle joints were reported among the included studies. The knee joint articular cartilage / osteochondral defects were located on the medial femoral condyle (*n* = 291), lateral femoral condyle (*n* = 115), femoral condyles without side specification (*n* = 814), patella (*n* = 175), trochlea (*n* = 10), tibial condyles (*n* = 47). The ankle joint articular cartilage / osteochondral defects were located in the talar dome: medial (*n* = 95); lateral (*n* = 45); both medial and lateral (*n* = 1); central (*n* = 3); anteromedial (*n* = 10); anterocentral (*n* = 2); anterolateral (*n* = 3); centromedial (*n* = 7); central direct (*n* = 1); centrolateral (*n* = 17) and posteromedial (*n* = 48). One of the studies did not report the defect location (Hangody et al. 2001a). The defect’s sizes averages ranged from 1.0 cm^2^ to 4.9 cm^2^ for the knee and 0.85 cm^2^ to 1.8 cm^2^ for the ankle joint.

### Surgical procedure

Table 3 depicts the characteristics of the mosaicplasty procedures and the subsequent radiological outcomes. The duration of symptoms until the time of surgery was poorly reported for the knee joint, since only two studies (Gudas et al. 2005; Clavé et al. 2016) reported this parameter (21.3 and 66.1 months). The reported duration of symptoms for the ankle joint ranged between 9 and 50.4 months (Al-Shaikh et al. 2002; Baltzer & Arnold 2005; de l'Escalopier et al. 2015; Hangody et al. 2001a; Kim et al. 2012; Lee et al. 2003). The reported follow-up duration ranged from 12 to 115 months (Hangody et al. 2010; Espregueira-Mendes et al. 2012; Gudas et al. 2005; Atik et al. 2005; Jakob et al. 2002; Kock et al. 2010; Koulalis et al. 2004; Quarch et al. 2014; Reverte-Vinaixa et al. 2013; Clavé et al. 2016) for the knee mosaicplasty and from 6 to 76 months (Reddy et al. 2007; Ahmad & Jones 2015; Al-Shaikh et al. 2002; Baltzer & Arnold 2005; de l'Escalopier et al. 2015; Gautier et al. 2002; Hangody et al. 2001a; Kim et al. 2012; Lee et al. 2003; Valderrabano et al. 2009) for the ankle mosaicplasty.

**Table 3:** Mosaicplasty surgical procedure characteristics and outcomes

First author (year)	Donor site	Plugs size (mm)	No. plugs (range)	Time to surgery (months)	No. of previous surgeries	No. concomitant procedures	Follow-up (months)	Radiological outcomes	Satisfaction (%)	Return to sports activity (%)	Second-look arthroscopy (%)	Complications
Knee												
Atik et al. (2005)	Minimal weightbearing area of the patellofemoral joint or the intercondylar notch area	3.5	≤5	N.R.	N.R.	N.R.	48	Normal shiny appearance and color of the grafted area (100 %)^a^	N.R.	N.R.	42	Slight joint effusion (*n* = 12).
Espregueira-Mendes et al. (2012)	Upper tibio-fibular joint	N.R.	2.5 (1-6)	N.R.	N.R.	N.R.	110.1	MRI-scoring system: good (26 %), fair (65 %) and poor (10 %)^d^	90	N.R.	N.R.	N.R.
Gudas et al. (2005)	Lateral/medial margin of the femoral trochlea	5.5	4.3 (3-6)	21.3	0	N.R.	37.1	ICRS: 27 (96 %) good to excellent results.^cd^	N.R.	93	50	Superficial infection (*n* = 2).
Hangody et al. (2008)	Margin of the medial and lateral femoral condyle superior to the sulcus terminalis and notch area (for larger defects)	N.R.	N.R.	N.R.	N.R.	783	At least 12	N.R.	N.R.	N.R.	10	Deep infections (*n* = 4), painful haemarthroses (*n* = 56), minor thromboembolic complications (*n* = 4).
Hangody et al. (2010)	Margin of the medial and lateral femoral condyle superior to the sulcus terminalis	4.5-8.5	2.7 (1-9)	N.R.	N.R.	225	115.2	Fairbank: grades I-II in 19 % and grades II-III in 8%^c^	90	91	7	Septic arthritis (*n* = 2), intra-articular hemorage (*n* = 2).
Jakob et al. (2002)	Medial and the lateral edging of the femoral trochlea and notch area (for larger defects)	6.3	6 (1-16)	N.R.	3	47	37	ICRS: nearly normal (grade II) in 91%^d^ Demarcating border (<1 mm) between the grafted surface and the surrounding cartilage^d^ Smooth or slightly fibrillated surface of autografts^d^	88	52	24	Reflex sympathetic dystrophy (*n* = 1), graft failure (*n* = 4), severe infection (*n* = 1) and postoperative joint stiffness (*n* = 1).
Kock et al. (2010)	Trochlear border of the lateral femoral condyle	9.4	2.4 (1–4)	N.R.	N.R.	N.R.	49	Did not performed radiological evaluation	N.R.	N.R.	N.R.	N.R.
Koulalis et al. (2004)	Lateral femoral condyle	2.9	2.9 (1–7)	N.R.	N.R.	3	27.2	ICRS: normal (67 %) and nearly normal (33 %)^d^ Cartilaginous coverage of the defect (100 %)^d^ Osseous integration of the grafts (100 %)^d^	N.R.	N.R.	22	N.R.
Quarch et al. (2014)	Dorsal medial femoral condyle	8.5	3.8	N.R.	N.R.	N.R.	13.8	Henderson score (modified): 11.1 points^d^	N.R.	N.R.	N.R.	Bone marrow edema with osseous cysts (*n* = 1).
Reverte-Vinaixa et al. (2013)	Lateral edge of the trochlea	9.3	2.8 (1-7)	N.R.	12	N.R.	12	Surface congruency and correct graft integration with no signs of fissuring or delamination (88 %)^d^	N.R.	N.R.	N.R.	Necrosis and cystic degeneration of the grafts (*n* = 2) and persistent graft oedema (*n* = 5).
Clavé et al. (2016)	Trochlear facets/groove and intercondylar notch	8.6	1.6 (1-4)	66.1	13	N.R.	24	N.R.	N.R.	N.R.	N.R.	Intra-articular effusion (*n* = 2); hematoma (*n* = 1); popliteal cyst (*n* = 1).
Ankle												
Ahmad and Jones (2015)	Extra-articular superolateral distal femoral condyle	N.R.	N.R.	N.R.	N.R.	N.R.	35.2	Full osteochondral healing (90 %)^c^	N.R.	N.R.	N.R.	*Post*-operative superficial wound blistering (*n* = 1) and non-union graft (*n* = 2).
Al-Shaikh et al. (2002)	Trochlear border of the lateral femoral condyle	8.6	1.3 (1-2)	50.4	13	1	16	No evidence of graft subsidence and all grafts healed (100 %)^c^	83	N.R.	N.R.	Neuroma (*n* = 2); superficial wound slough (*n* = 1); symptomatic hardware (*n* = 1).
Baltzer and Arnold (2005)	Superolateral condyle of the ipsilateral knee	N.R.	1.8 (1-4)	>9	N.R.	N.R.	Up to 54	Bone integration into the talus^cd^	95	Most of included participants	At least 58	N.R.
Gautier et al. (2002)	Non-weight-bearing trochlear border of the ipsilateral knee	6.4	4.4 (1-6)	N.R.	N.R.	N.R.	24	Incorporation of the graft and intergraft intergration (91 %)^e^	91	100	27	Partial resorption of graft (*n* = 1)
Hangody et al. (2001a), (2001b)	Minimal weightbearing areas of the femoral condyles at the level of the patellofemoral joint	4.5-6.5 (*n* = 27) 3.5 (*n* = 9)	3 (1-6)	9	29	N.R.	50.4	Good incorporation of all the transplanted grafts^c^	N.R.	N.R.	22	None
Kim et al. (2012)	Lateral edge of the lateral trochlea	9.3	1.4 (1-2)	20.4	10	14	34.1	Congruent graft margins (88 %)^a^	95	N.R.	100	Adhesion (*n* = 15); synovitis (*n* = 16); incongruent surface of the graft (*n* = 10); uncovered area (*n* = 14).
Lee et al. (2003)	Superomedial margin (nonweightbearing area) of the medial femoral condyle of the ipsilateral knee	6-7	2.2 (2-4)	12.2	N.R.	N.R.	36	Consistency of articular surface of the grafts and congruity between the native cartilage (88 %)^a^	N.R.	76	94	N.R.
de l'Escalopier et al. (2015)	Medial (*n* = 15) and lateral (*n* = 22) edge of the trochlea	5.7	2.3 (1-8)	29	8	8	76	Joint space narrowing (16 %)	N.R.	N.R.	N.R.	None
Reddy et al. (2007)	Intercondylar notch or the lateral femoral condyle proximal to the sulcus terminale	5.0	2.9 (2-4)	N.R.	N.R.	N.R.	47	N.R.	82	N.R.	N.R.	N.R.
Valderrabano et al. (2009)	Lateral femoral condyle	N.R.	3 (2-6)	N.R.	>9	N.R.	72	Partially narrowed cartilage (100 %), no joint space narrowing (67 %) and subchondral bone plate was partially disrupted (58 %) or missing (25 %)b^d^	92	50	N.R.	Cyst formation (*n* = 11); bone bruising (*n* = 9); loose bodies (*n* = 5). ^b^

*Legends: ICRS* International Cartilage Repair Society score, *N.R.* Not reported, *MRI* Magnetic resonance imaging. Footnotes: ^a^Results reported through second-look arthroscopy; ^b^Report of the 12 included patients on the follow-up, from a cohort of 21 patients; ^c^ Results reported through radiography; ^d^ Results reported through MRI; ^e^ Results reported through CT

The donor-sites used for the osteochondral graft harvesting varied across the studies, including: margins of the medial femoral trochlea (condyle) (Hangody et al. 2010; Gudas et al. 2005; Ahmad & Jones 2015; de l'Escalopier et al. 2015; Gautier et al. 2002; Hangody et al. 2001a; Hangody et al. 2008; Jakob et al. 2002; Lee et al. 2003; Quarch et al. 2014); margins of the lateral femoral trochlea (condyle) (Hangody et al. 2010; Reddy et al. 2007; Gudas et al. 2005; Ahmad & Jones 2015; Al-Shaikh et al. 2002; Baltzer & Arnold 2005; de l'Escalopier et al. 2015; Gautier et al. 2002; Hangody et al. 2001a; Hangody et al. 2008; Jakob et al. 2002; Kim et al. 2012; Kock et al. 2010; Koulalis et al. 2004; Reverte-Vinaixa et al. 2013; Valderrabano et al. 2009); minimal weight-bearing areas of the patellofemoral joint (Atik et al. 2005); intercondylar notch area (Reddy et al. 2007; Atik et al. 2005; Hangody et al. 2008); upper tibio-fibular joint (Espregueira-Mendes et al. 2012).

Regarding the number of plugs used in each mosaicplasty and their size, both varied across the studies. When considering the number of plugs, the majority of the studies used 3 or less plugs per each mosaicplasty procedure, either for the knee (6/11) (Hangody et al. 2010; Espregueira-Mendes et al. 2012; Kock et al. 2010; Koulalis et al. 2004; Reverte-Vinaixa et al. 2013; Clavé et al. 2016) or ankle joints (8/10) (Reddy et al. 2007; Al-Shaikh et al. 2002; Baltzer & Arnold 2005; de l'Escalopier et al. 2015; Hangody et al. 2001a; Kim et al. 2012; Lee et al. 2003; Valderrabano et al. 2009). The remaining studies reported more than 3 plugs used per patient (Gudas et al. 2005; Atik et al. 2005; Gautier et al. 2002; Jakob et al. 2002; Quarch et al. 2014) or did not stated the number of plugs used (Ahmad & Jones 2015; Hangody et al. 2008). Concerning the plugs size, for the knee joint the plugs’ size averages ranged from 2.9 to 9.4 mm (Hangody et al. 2010; Gudas et al. 2005; Atik et al. 2005; Jakob et al. 2002; Kock et al. 2010; Koulalis et al. 2004; Quarch et al. 2014; Reverte-Vinaixa et al. 2013; Clavé et al. 2016), and for the ankle joint from 3.5 to 9.3 mm (Reddy et al. 2007; Al-Shaikh et al. 2002; de l'Escalopier et al. 2015; Gautier et al. 2002; Hangody et al. 2001a; Kim et al. 2012; Lee et al. 2003). Five studies (Espregueira-Mendes et al. 2012; Ahmad & Jones 2015; Baltzer & Arnold 2005; Hangody et al. 2008; Valderrabano et al. 2009) did not made any report on the plugs size.

The description of the surgeries performed previously to the mosaicplasty procedure was also poorly reported. A combined number of 26 and 69 previous surgical procedures were reported for the knee and ankle joints, respectively. Regarding the knee joint, the previous reported surgical procedures included high tibial opening wedge osteotomies (Jakob et al. 2002), unspecified osteotomies, meniscectomy, ACL reconstruction, microfracture, Pridie drilling, diagnostic arthroscopy, and cartilage shaving / debridement (Reverte-Vinaixa et al. 2013; Clavé et al. 2016). In turn¸ Gudas et al. (Gudas et al. 2005) reported that their patients had never underwent any surgical procedure on the affected knee. Regarding the ankle joint, previous surgical procedures to the mosaicplasty include failed excision, curettage, arthroscopic debridement, osteosynthesis, implant and/or fragment removal, exploratory arthroscopy, loose body removal, retrograde or anterograde drilling, microfracture and subchondral perforation procedures (Al-Shaikh et al. 2002; de l'Escalopier et al. 2015; Hangody et al. 2001a; Kim et al. 2012; Valderrabano et al. 2009).

Overall, a total of 1058 and 23 concomitant surgeries were reported along the knee and ankle mosaicplasty procedures, respectively. Concomitant surgeries performed during the knee mosaicplasty procedure included ACL reconstruction, realignment osteotomies, meniscus surgery, patellofemoral realignment procedures, lateral retinacular release, tibial turbercle transplantation, trochleoplasty, reconstruction of the lateral collateral ligament and total synovectomy (Hangody et al. 2010; Hangody et al. 2008; Jakob et al. 2002; Koulalis et al. 2004). Concerning the ankle mosaicplasty procedure, the reported concomitant surgeries comprised the modified Broström operation, cancellous bone grafting between the plugs, subchondral bone perforation, removal of a free intra-articular osteochondral fragments and lateral ankle ligament reconstruction (de l'Escalopier et al. 2015; Kim et al. 2012). In addition, one patient presented a lateral meniscus tear during the articular cartilage harvesting and therefore, a partial meniscectomy was performed (Al-Shaikh et al. 2002).

Regarding the surgical complications, a combined number of 192 complications were reported, 104 related to the knee mosaicplaty procedure (Hangody et al. 2010; Gudas et al. 2005; Atik et al. 2005; Hangody et al. 2008; Jakob et al. 2002; Quarch et al. 2014; Reverte-Vinaixa et al. 2013; Clavé et al. 2016) and 88 related to the ankle mosaicplasty procedure (Ahmad & Jones 2015; Al-Shaikh et al. 2002; Gautier et al. 2002; Kim et al. 2012; Valderrabano et al. 2009). A more detailed depiction of the complications is described in Table 3. In addition, the radiological outcomes, satisfaction and return to sports rates are summarized in Table 3.

### Donor-site morbidity

The reported figures of knee donor-site morbidity varied considerably across the included studies and its description is depicted in Table 4. In this sense, there were 4 studies reporting no donor-site morbidity (Espregueira-Mendes et al. 2012; Gudas et al. 2005; Atik et al. 2005; Kim et al. 2012), 10 studies reporting donor-site morbidity in less than 20 % of their cohort (Hangody et al. 2010; Baltzer & Arnold 2005; de l'Escalopier et al. 2015; Hangody et al. 2001a; Hangody et al. 2008; Lee et al. 2003; Quarch et al. 2014; Reverte-Vinaixa et al. 2013; Valderrabano et al. 2009; Clavé et al. 2016) and, 3 studies of knee-to-knee (Jakob et al. 2002; Kock et al. 2010; Koulalis et al. 2004) and 4 studies of knee-to-ankle (Reddy et al. 2007; Ahmad & Jones 2015; Al-Shaikh et al. 2002; Gautier et al. 2002) mosaicplasty surgical procedures reported donor-site associated morbidity in over than 35 % of their cohort.

**Table 4:** Knee donor site related morbidity description and percentages

	First author (year)	Donor site morbidity	Percentage of morbidity
Knee	Atik et al. (2005)	None	0 %
	Espregueira-Mendes et al. (2012)	None	0 %
	Gudas et al. (2005)	None	0 %
	Hangody et al. (2008)	Moderate and severe donor site disturbances (*n* = 29)	3 %
	Hangody et al. (2010)	Patellofemoral complaints (*n* = 15)	5 %
	Jakob et al. (2002)	Minor postoperative effusion (*n* = 1)	38 %
		Crepitation (*n* = 15)	
	Kock et al. (2010)	Retropatellar crepitus (*n* = 12)	92 %
	Koulalis et al. (2004)	Patellar chondropathy (*n* = 4) and joint effusion (*n* = 7)	39 %
	Quarch et al. (2014)	Discomfort on the back of the knee during stair climbing or kneeling (*n* = 2)	13 %
	Reverte-Vinaixa et al. (2013)	Osteoarthritis (*n* = 1)	6 %
	Clavé et al. (2016)	Persistent patellofemoral pain (*n* = 1)	4 %
Ankle	Ahmad and Jones (2015)	Knee stiffness or “catching and popping” (*n* = 6)	45 %
		Moderate knee pain and swelling after prolonged standing and walking (*n* = 2)	
		Moderate to severe knee pain, swelling, and stiffness after moderate weightbearing activities (*n* = 1)	
	Al-Shaikh et al. (2002)	Pain during severe exertion (*n* = 7)	42 %
		Giving-way and knee swelling symptoms (*n* = 1)	
	Baltzer and Arnold (2005)	Donor site disturbances (*n* = 1)	2 %
	Gautier et al. (2002)	Mild pain walking down stairs (*n* = 1)	55 %
		Mild to moderate difficulty on kneeling (*n* = 2)	
		Mild difficulty on squatting and jumping (*n* = 2)	
		Mild stiffness after strenuous activity (*n* = 1)	
	Hangody et al. (2001a), (2001b)	Slight to moderate pain in the patellofemoral area during strenuous physical activity (*n* = 6)	17 %
	Kim et al. (2012)	None	0 %
	Lee et al. (2003)	Mild soreness, mild aching, and some crepitus when flexing the knee (*n* = 2)	12 %
	de l'Escalopier et al. (2015)	Persistent knee pain (*n* = 6)	16 %
	Reddy et al. (2007)	Instability in daily activities, pain after walking a mile or more, having a slight limp, and difficulty squatting (*n* = 6)	54 %
	Valderrabano et al. (2009)	Recurrent joint swelling (*n* = 1)	17 %
		Giving-way symptoms (*n* = 1)	

**Fig. 2:**
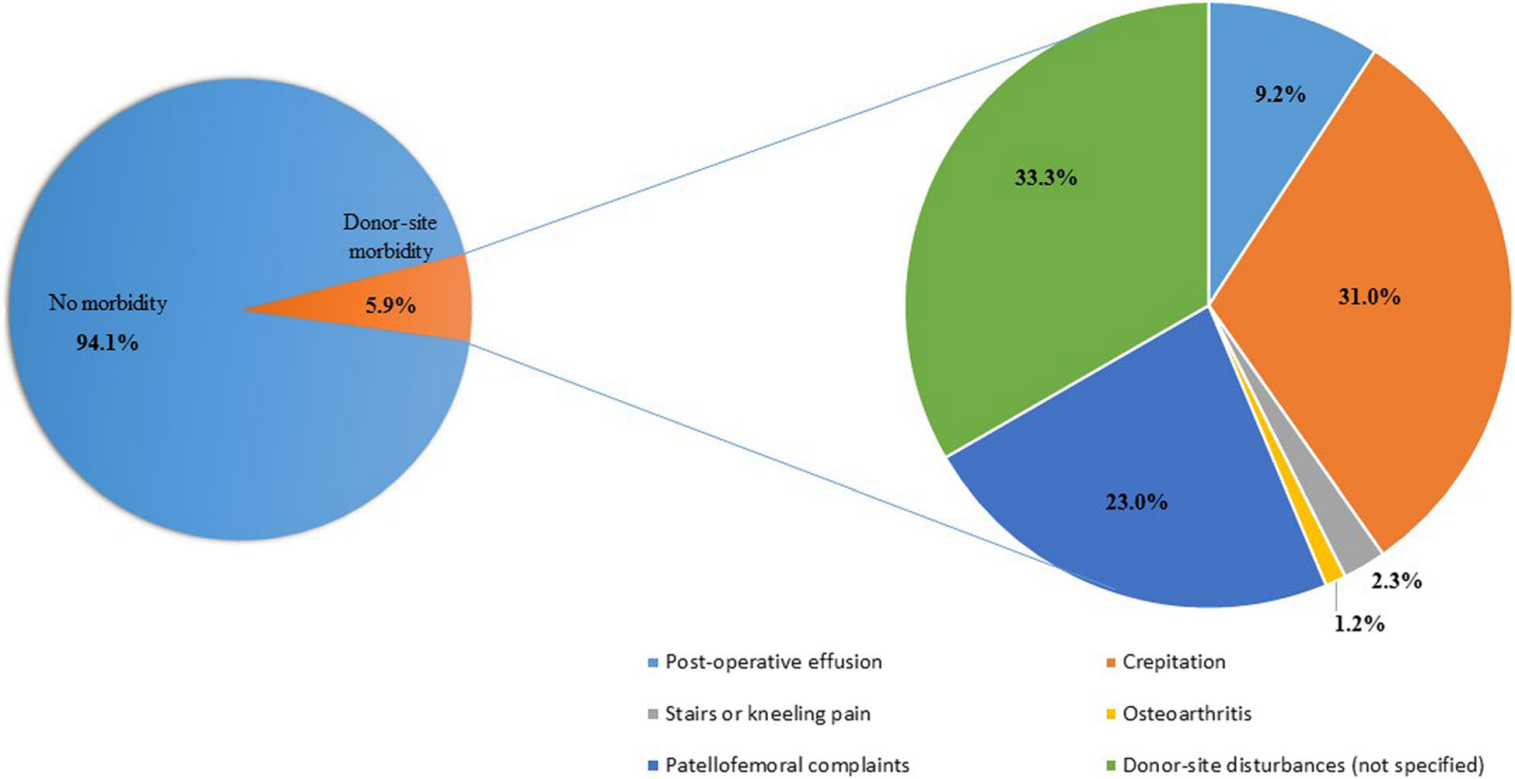
Knee donor-site morbidity figures from knee-to-knee mosaicplasty procedure

**Fig. 3:**
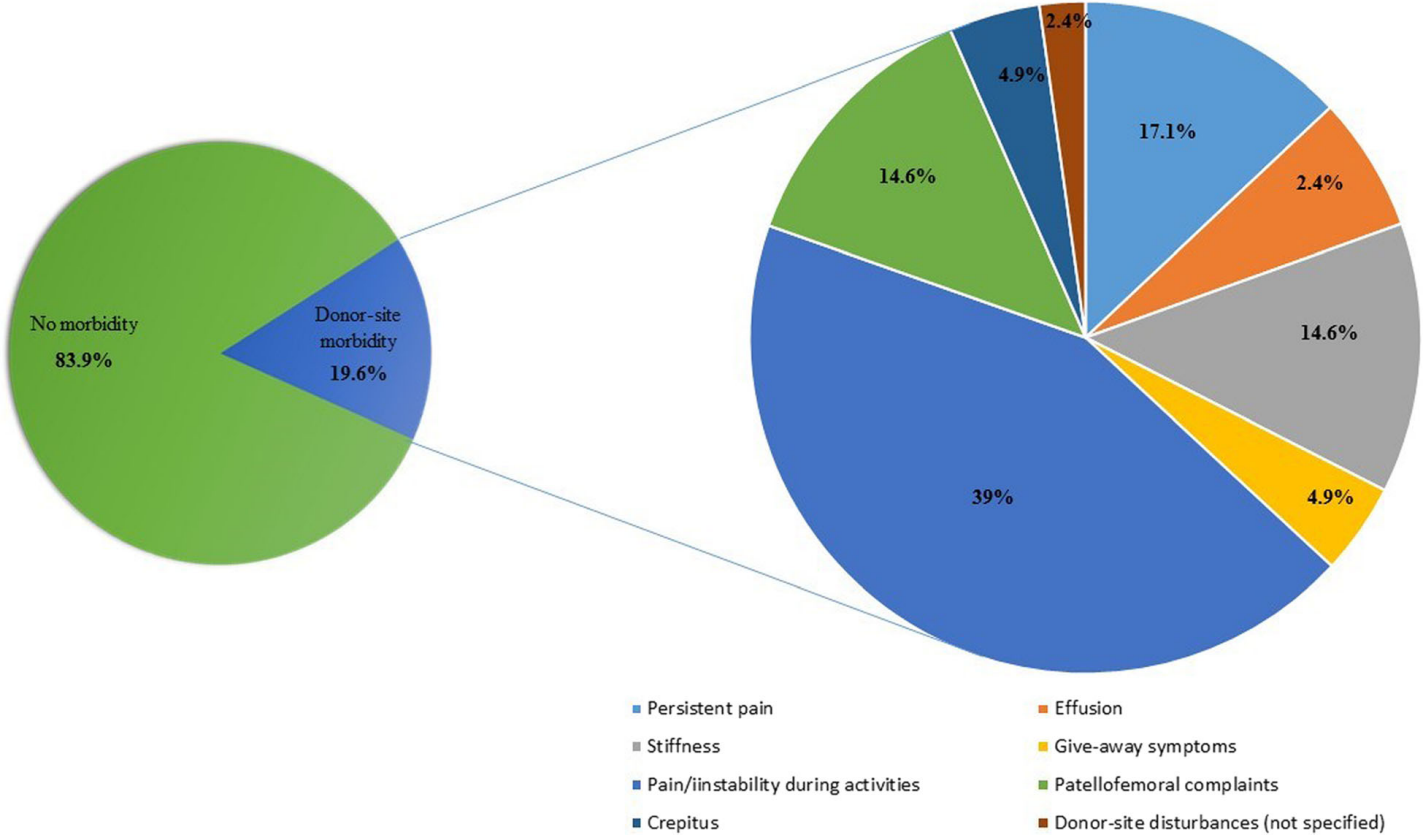
Knee donor-site morbidity figures from knee-to-ankle mosaicplasty procedure

Overall, the studies reported donor-site morbidity rates ranging from 0 % to 92 % (knee-to-knee) and 0 % to 55 % (knee-to-ankle), with pooled rates of 5.9 % and 19.6 % regarding the knee donor-site associated morbidity after knee (Fig. 2) and ankle (Fig. 3) mosaicplasty, respectively. In knee-to-knee mosaicplasty, the most common donor-site morbidity complaints were patellofemoral disturbances (23 %) (Hangody et al. 2010; Koulalis et al. 2004; Clavé et al. 2016) and crepitation (31 %) (Jakob et al. 2002; Kock et al. 2010). Post-operative effusion was reported in 9 % of the patients (Jakob et al. 2002; Koulalis et al. 2004) and 33 % did not specified their complaints (Hangody et al. 2008). Regarding the knee-to-ankle mosaicplasty procedures, the most prevalent knee donor-site complaint reported was pain or instability during daily living or sports activities (44 %) (Ahmad & Jones 2015; Al-Shaikh et al. 2002; Gautier et al. 2002). In addition, patellofemoral disturbances (13 %) (Hangody et al. 2001a), knee stiffness (13 %) (Ahmad & Jones 2015) and persistent pain (13 %) (Reddy et al. 2007; de l'Escalopier et al. 2015) were also common complaints.

**Fig. 4:**
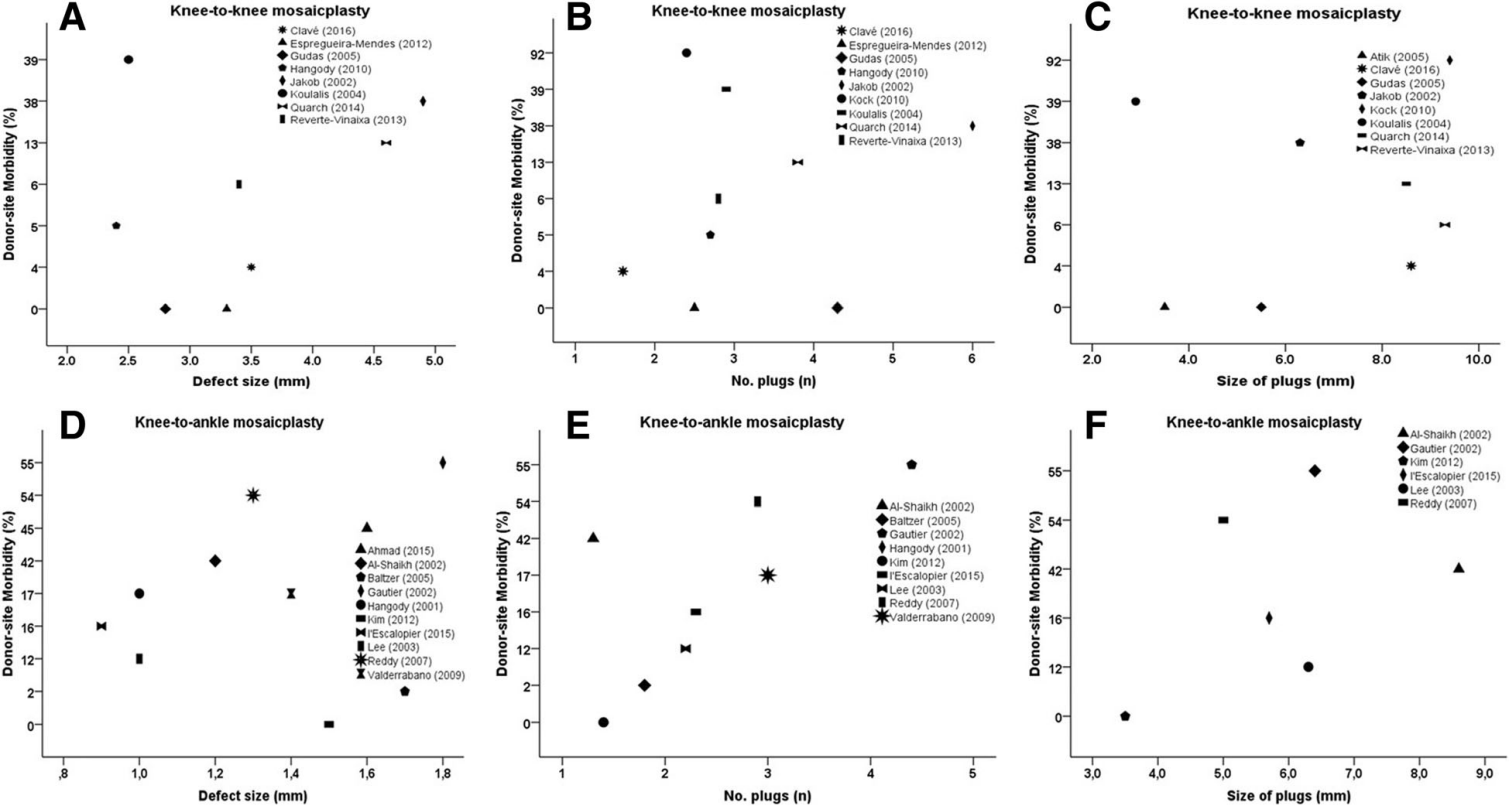
Correlation between the donor-site morbidity rate (%) from mosaicplasty harvesting and mean defect size (mm), mean number of plugs (n) and mean size of plugs (mm). **a**) Knee-to-knee mosaicplasty donor-site morbidity rate (%) vs mean defect size (mm), (*r* = 0.228, *p* = 0.588); **b**) Knee-to-knee mosaicplasty donor-site morbidity rate (%) vs mean number of plugs (mm), (*r* = -0.109, *p* = 0.781); **c**) Knee-to-knee mosaicplasty donor-site morbidity rate (%) vs mean size of plugs (mm), (*r* = 0.275, *p* = 0.509); **d**) Knee-to-ankle mosaicplasty donor-site morbidity rate (%) vs mean defect size (mm), (*r* = 0.216, *p* = 0.548); **e**) Knee-to-ankle mosaicplasty donor-site morbidity rate (%) vs mean number of plugs (mm), (*r* = 0.563, *p* = 0.114); **f**) Knee-to-ankle mosaicplasty donor-site morbidity rate (%) vs mean size of plugs (mm), (*r* = 0.486, *p* = 0.329)

Regarding the knee-to-knee mosaicplasty procedures, there were no significant correlations between the donor-site morbidity rate and mean defect size (*r* = 0.228, *p* = 0.588, *n* = 8), mean number of plugs (*r* = -0.109, *p* = 0.781, *n* = 9) and mean size of plugs (*r* = 0.275, *p* = 0.509, *n* = 8; Fig. 4). In the same line, the knee-to-ankle mosaicplasty procedures did not showed significant correlations between the donor-site morbidity rate and mean defect size (*r* = 0.216, *p* = 0.548, *n* = 10), mean number of plugs (*r* = 0.563, *p* = 0.114, *n* = 9) and mean size of plugs (*r* = 0.486, *p* = 0.329, *n* = 6).

### Methodological quality

The mean Coleman Methodology Score was 49.7 ± 15 points (Table 5) and all-but-two studies were classified as level III (Hangody et al. 2010; Reddy et al. 2007; de l'Escalopier et al. 2015; Hangody et al. 2008; Jakob et al. 2002; Kim et al. 2012; Quarch et al. 2014) or IV (Espregueira-Mendes et al. 2012; Ahmad & Jones 2015; Al-Shaikh et al. 2002; Atik et al. 2005; Baltzer & Arnold 2005; Gautier et al. 2002; Hangody et al. 2001a; Kock et al. 2010; Koulalis et al. 2004; Lee et al. 2003; Reverte-Vinaixa et al. 2013; Valderrabano et al. 2009). The exception was one level I (Clavé et al. 2016) and one level II study (Gudas et al. 2005).

**Table 5:** Methodological quality according Coleman Methodology Score

Coleman Methodology Score	Knee	Ankle	Total
	Mean (SD)	Mean (SD)	Mean (SD)
Part A			
Study size (10)	3.8 (4.1)	2.6 (3)	3.2 (3.6)
Mean duration follow-up (5)	3.9 (1.5)	3.9 (1.9)	3.9 (1.6)
No. of treatment procedures (10)	10 (0.0)	10 (0.0)	10 (0)
Type of study (15)	2.7 (6.1)	0 (0.0)	1.4 (4.5)
Diagnostic certainty (5)	5 (0.0)	5 (0.0)	5 (0.0)
Description of surgical procedure (5)	4.3 (1)	4.6 (0.8)	4.4 (0.9)
Rehabilitation & compliance (10)	6.4 (5.0)	5.0 (5.3)	5.7 (5.1)
Part B			
Outcome criteria (10)	8.9 (1.4)	7.5 (1.7)	8.2 (1.7)
Outcome assessment (15)	6 (4.8)	5.1 (4.4)	5.6 (4.5)
Selection process (15)	2.1 (4.7)	2.3 (2.5)	2.2 (3.7)
Total part A (60)	36.1 (11.2)	31.1 (7.4)	33.7 (9.7)
Total part B (40)	17 (8.4)	14.9 (5.6)	16 (7.1)
Total score (100)	53.1 (18.3)	46 (10.1)	49.7 (15.0)
No. studies (%)			
Level I	1 (9)	0 (0)	1 (5)
Level II	1 (9)	0 (0)	1 (5)
Level III	4 (36)	3 (30)	7 (33)
Level IV	5 (46)	7 (70)	12 (57)

Several major issues were found for both knee-to-knee and knee-to-ankle mosaicplasty procedure. The type of study was poorly scored and included small sample sizes (*n* < 20) (Reddy et al. 2007; Al-Shaikh et al. 2002; Atik et al. 2005; Gautier et al. 2002; Kock et al. 2010; Koulalis et al. 2004; Lee et al. 2003; Quarch et al. 2014; Reverte-Vinaixa et al. 2013; Valderrabano et al. 2009). In addition, the procedure for assessing the outcomes (Reddy et al. 2007; Al-Shaikh et al. 2002; Atik et al. 2005; Baltzer & Arnold 2005; de l'Escalopier et al. 2015; Gautier et al. 2002; Hangody et al. 2001a; Hangody et al. 2008; Jakob et al. 2002; Kim et al. 2012; Koulalis et al. 2004; Lee et al. 2003; Quarch et al. 2014) and the description of the subject selection process (Hangody et al. 2010; Espregueira-Mendes et al. 2012; Reddy et al. 2007; Ahmad & Jones 2015; Al-Shaikh et al. 2002; Atik et al. 2005; Baltzer & Arnold 2005; de l'Escalopier et al. 2015; Gautier et al. 2002; Hangody et al. 2001a; Hangody et al. 2008; Jakob et al. 2002; Kim et al. 2012; Kock et al. 2010; Koulalis et al. 2004; Lee et al. 2003; Quarch et al. 2014; Reverte-Vinaixa et al. 2013; Valderrabano et al. 2009; Kreuz et al. 2006) were also poorly reported across the included original studies.

## Discussion

The main findings of this systematic review show that harvesting osteochondral plugs from the knee joint often results in considerable donor-site morbidity for knee-to-knee (5.9 %) and knee-to-ankle (16.9 %) mosaicplasty procedures. The higher percentage of morbidity for knee-to-ankle procedures in regard to the knee-to-knee procedures may be related to the higher number of knee patients (*n* = 1472 vs. *n* = 254). Moreover, in the knee-to-knee mosaicplasty procedures, patients may have lived with knee pain for long periods of time, coping better with knee symptomatology, increasing their tolerance to pain and decreasing their outcome expectations. Additionally, it is possible that the eventual difficulty in attributing the post-operative knee morbidity to the donor-site may also have lowered rate in the knee-to-knee procedures. These results highlight the importance of finding a surgical alternative that is able to correct and address the articular defect without leading to iatrogenic hazard.

Regarding the number of plugs used in each mosaicplasty procedure, most of the studies used in average 3 or less plugs with a considerable variability in the size of the plugs. In this sense, smaller-sized plugs may be suitable to fill irregular cartilage defects with lower donor-site morbidity expected. Nevertheless, smaller grafts are known to be more fragile, with lower pullout strength and more technically demanding (Kordás et al. 2005). Still, no significant correlation was found between the number and size of plugs and the donor-site morbidity rate (*p* > 0.05).

The osteochondral grafts were harvested mostly from the margins of the medial and/or lateral femoral trochlea (condyle). Nevertheless, other donor-site sources within the knee were reported, including the minimal weight-bearing areas of the patellofemoral joint (Atik et al. 2005); intercondylar notch area (Reddy et al. 2007; Atik et al. 2005; Hangody et al. 2008); upper tibio-fibular joint (Espregueira-Mendes et al. 2012). After analysis of the included studies reported donor-site morbidity, the patellofemoral joint (Atik et al. 2005) and the upper tibio-fibular joint (Espregueira-Mendes et al. 2012) seem to be reasonable donor-sites to harvest osteochondral plugs without any associated morbidity.

Although good results are being reported in the scientific literature regarding the mosaicplasty procedures, there is still the need to bear in mind the potential donor-site morbidity arising from the osteochondral plugs harvesting. In fact, when pooling the donor-site morbidity rates reported in the literature, the figures range substantially from 0 to 92 % and 0 to 55 %, with calculated pooled rates of 5.9 and 19.6 % for the knee and ankle joint, respectively. This illustrates the conflicting evidence within the scientific literature regarding the potential risks for the donor-site after harvesting. In addition, donor-site morbidity from knee-to-knee mosaicplasty procedures resulted essentially in pain and mechanical symptoms. On the other hand, harvesting osteochondral grafts from the knee to transplant to the ankle joint led mostly to persistent pain and instability. Reports of fibrocartilage hypertrophy (LaPrade & Botker 2004), loose bodies (Kim & Shin 2000) and bony lesions (Nakagawa et al. 2005) have also been found in the scientific literature. In this sense, the surgeon should be aware these potential donor-site morbidity risks while planning the mosaicplasty surgery.

The donor-site associated morbidity after mosaicplasty is seldom properly described and evaluated in the scientific literature. The healing processes at the donor-site after the graft harvesting are made through a creeping ingrowth of autogenous cancellous bone and an overlying fibrocartilage-like cover into the donor holes (Bedi et al. 2010; Tytherleigh-Strong & Miniaci 2003; Feczkó et al. 2003). Recent reports of filling the donor holes with biocompatible material have been published (Feczkó et al. 2003; Bartha et al. 2013), aiming to reduce the donor-site morbidity after the osteochondral harvesting. Nevertheless, the best approach may be to preserve the weight-bearing areas of the knee joint and harvest the osteochondral plugs from potential morbidity-free, minimal non-weight-bearing areas. In this sense, several alternative donor-site areas for mosaicplasty harvesting have been proposed. While the posterior femoral condyles and the calcaneal tuberosity cartilage were considered as unsuitable donor-site alternatives for osteochondral autografting (Calder et al. 2015; Thaunat & Beaufils 2010), the lower weight-bearing area of the patellofemoral joint and the upper tibio-fibular joint showed promising results in humans without donor-site morbidity associated (Espregueira-Mendes et al. 2012; Atik et al. 2005).

### Limitations

This systematic review has its inherent limitations related to this type of study. Conclusions are limited by the quality of the studies available for inclusion. In this sense, this systematic review included mostly level IV studies (Espregueira-Mendes et al. 2012; Ahmad & Jones 2015; Al-Shaikh et al. 2002; Atik et al. 2005; Baltzer & Arnold 2005; Gautier et al. 2002; Hangody et al. 2001a; Kock et al. 2010; Koulalis et al. 2004; Lee et al. 2003; Reverte-Vinaixa et al. 2013; Valderrabano et al. 2009) and there is an obvious lack for studies with higher level of evidence. Moreover, a low Coleman Methodological Score (mean 48 of 100 possible) was also verified.

In addition, the major limitation found was the lack of morbidity data reported within the original studies. Most of the studies did not described or even reported the donor-site morbidity associated with the graft harvesting, which could had led to reporting bias and under- or overestimation of the problem. The lack of reporting of donor-site morbidity data is more frequent in the knee-to-knee mosaicplasty studies. In this sense, it would be useful to have comprehensive reports regarding donor-site morbidity in related future publications. The quantification of donor sites used and their correlation with the occurrence of associated morbidity was one of the main end-points intended, however this was not possible since the original studies often report more than one donor site without reporting how many patients were allocated to each donor-site. In addition, the lack of control/comparison groups and objective quantification of the donor-site morbidity within the original studies did not allowed the performance of a more systematic quantitative analysis (meta-analysis).

Another concern was the overlapping of cohorts or subgroups of cohorts in longitudinal long-term follow-up studies (Hangody et al. 2010; Hangody & Füles 2003; Hangody et al. 2001a; Hangody et al. 2008; Hangody et al. 2001b; Szerb et al. 2005), an increasingly concern in the orthopaedics and surgery scientific literature (Jakobsen et al. 2005; Gwilym et al. 2004; Schein & Paladugu 2001). Concerning this issue, studies with biggest cohort and longest follow-up were selected (Hangody et al. 2010; Hangody et al. 2008; Hangody et al. 2001b).

Finally, it was not found any correlation between the defect size, number and size of the plugs and the rate of donor-site morbidity. A potential correlation might have been cloaked by the wide range of donor-site morbidity rates reported among the studies, different surgical techniques, chosen donor-site for harvesting and small sample sizes. Additionally, the low number of studies eligible for the statistical analysis may have increased the risk for type 2 error.

The rate of donor-site morbidity is quite high and maybe not acceptable. However, since better treatment options are currently lacking, surgeons have to deal with it. In order to improve the harvesting procedure and lower its related morbidity, future studies should improve the quality of reporting data on donor-site morbidity. In this sense, the authors propose a donor-site morbidity evaluation protocol including consistent time assessment points (eg., at 1, 3, 6, 12 and 24 months), including pre and post-operative assessment with specific patellofemoral outcome score (eg., Lysholm and Kujala scores) and a post-operative magnetic resonance imaging for assessing possible progression of bone and cartilage damage in the donor-site area.

## Conclusion

The donor-site morbidity for knee-to-ankle (16.9 %) was greater than knee-to-knee (5.9 %) mosaicplasty procedures. While in knee-to-knee mosaicplasty, the most common donor-site morbidity complaints were patellofemoral disturbances (22 %) and crepitation (31 %), in knee-to-ankle there was a clear tendency for pain or instability during daily living or sports activities (44 %), followed by patellofemoral disturbances, knee stiffness and persistent pain (13 % each). Moreover, there was no significant correlation between rate of donor-site morbidity and size of the defect, number and size of the plugs.

## Abbreviations

ACL: Anterior cruciate ligament
CINHAL: Cumulative Index of Nursing and Allied Health
PRISMA: Preferred Reporting Items for Systematic Reviews and Meta-Analyses
PROSPERO: Prospective Register of Systematic Reviews
